# Educational interventions to improve the effectiveness in clinical competence of general practitioners: problem-based versus critical reading-based learning

**DOI:** 10.1186/1472-6920-12-53

**Published:** 2012-07-11

**Authors:** Javier Gongora-Ortega, Yolanda Segovia-Bernal, J de Jesus Valdivia-Martinez, J Martin Galaviz-deAnda, Carlos A Prado-Aguilar

**Affiliations:** 1 Unidad de Investigación en Salud, Instituto de Salud del Estado de Aguascalientes (ISEA), Aguascalientes, Ags, Mexico; 2 Unidad de Investigación Epidemiológica y en Servicios de Salud, Hospital General de Zona N 1, Instituto Mexicano del Seguro Social, Aguascalientes, Ags, Mexico; 3 Hospital General de Zona No. 2, Instituto Mexicano del Seguro Social, Aguascalientes, Ags, Mexico; 4 Depto. de Salud Publica, Universidad Autónoma de Aguascalientes, Aguascalientes, Ags, Mexico

## Abstract

**Background:**

Evidence suggests that continuing medical education improves the clinical competence of general practitioners and the quality of health care services. Thus, we evaluated the relative impact of two educational strategies, critical reading (CR) and problem based learning (PBL), on the clinical competence of general practitioners in a healthcare system characterized by excessive workload and fragmentation into small primary healthcare centers.

**Methods:**

Clinical competence was evaluated in general practitioners assigned to three groups based on the educational interventions used: 1) critical reading intervention; 2) problem based learning intervention; and 3) no intervention (control group, which continued clinical practice as normal). The effect on the clinical competence of general practitioners was evaluated in three dimensions: the cognitive dimension, via a self-administered questionnaire; the habitual behavioral dimension, via information from patient’s medical records; and the affective dimension, through interviews with patients. A paired Student´s t-test was used to evaluate the changes in the mean clinical competence scores before and after the intervention, and a 3 x 2 ANOVA was used to analyze groups, times and their interaction.

**Results:**

Nine general practitioners participated in the critical reading workshop, nine in the problem-based learning workshop, and ten were assigned to the control group. The participants exhibited no significant differences in clinical competence measures at baseline, or in socio-demographic or job characteristics (p > 0.05). Significant improvements in all three dimensions (cognitive, 45.67 vs 54.89; habitual behavioral, 53.78 vs 82.33; affective, 4.16 vs 4.76) were only observed in the problem-based learning group after the intervention (p > 0.017).

**Conclusions:**

While no differences in post-intervention scores were observed between groups, we conclude that problem-based learning can be effective, particularly in a small-group context. Indeed, problem-based learning was the only strategy to induce a significant difference between pre– and post- intervention scores for all three CC dimensions.

## Background

The Basic Package of Health Services (BPHS) is a set of strategies offered to all citizens by the government through federal and state participation, and it is government policy to provide universal access and coverage in response to priority health needs. This program offers a wide range of health services and involves low cost actions with high impact. The BPHS comprises 12 strategies that include 57 activities, 9 of which are performed by General Practitioners’ (GPs) at primary healthcare centers. In order to complete these activities with quality care, GPs must maintain an adequate level of clinical competence.

Clinical Competence (CC) reflects the ability of a physician to perform their activities in a healthcare setting when defining and managing a patient’s health problems, and it involves problem solving skills (*e.g.,* critical thinking and the application of clinical reasoning) and the ability to work as a team member and to communicate effectively [1].

CC involves three dimensions, the affective, cognitive and habitual behavior domains [2], although some authors refer to the affective dimension as “attitudes” [3]. CC has been defined as a pyramid with four parts: cognitive (what is known), competence (how it is known), performance (how it is proven) and actions (how it is done) [4]. Evaluating a physician’s CC provides them with feedback regarding their achievements and their limitations in detecting and resolving clinical problems within their sphere of influence. In this way, they can better target their educational activities to improve any deficiencies detected [1,5,6].

Different strategies have been used in Continuing Medical Education (CME) to influence and improve a physicians´ CC. One such strategy is collaborative active education (CAE) [7] or collaborative learning [8], which includes Problem-Based Learning (PBL), although the use of this technique in CME is limited [9,10]. PBL is considered a learning technique with great potential and it may be a key to improving medical practice. This type of learning involves an action-reflection-action process in which GPs identify problems from “clinical cases” or “diagnostic, therapeutic or malpractice controversies”, they search for new information to attain a better understanding of a problem, and they finally formulate a hypothesis to explain and ultimately solve the issue at hand. PBL helps GPs to resolve problems by stimulating discussion, dialogue, reflection and participation in the educational strategy [11]. This is the most comprehensive CME strategy as it takes in all the variables involved in a group learning process, and it provides GPs with a self-learning opportunity.

Another CME strategy is critical reading (CR). This is an active educational strategy that aims to improve in the ability of physicians to become aware of their position on a specific topic “almost automatically”. In CR, implied assumptions and core ideas are identified through the debate between physicians and the author, allowing the physicians to identify the strong and weak points of the main arguments in the text. In this way, the physician can propose alternative arguments that may improve upon the authors’ viewpoint, leading them to reaffirm or modify their own position. This strategy promotes an analysis of the text through a process of individual learning [12,13], and short learning courses have even been used previously as CME strategies [14]. Each of these strategies has produced mixed results and no single approach is clearly superior to others.

The effectiveness of PBL interventions in CME have been systematically reviewed [10]. While an analysis of 6 relevant studies (two randomized clinical trials and four clinical trials) provided limited evidence that PBL improves the knowledge and performance of GPs, or patient health, only 3 of these studies evaluated knowledge, performance and participant satisfaction, of which 2 revealed positive results in all three areas [11,15]. Moreover, in the third study the only positive effect was evident on participant satisfaction [16]. Contradictory results were obtained in two of these studies [11,16] that used different lecture-based interventions, neither of which clearly defined the learning process in the reading group.

We have yet to identify a study that compared the effects of PBL with those of CR intervention. PBL involves learning by guided discovery, while CR takes a repetition-based learning approach and significant learning improvements have been described for both strategies [17]. Thus, we sought to determine which of these educational strategies (CR or PBL) is more effective in improving physician CC in primary health services offered by the HISA in the city of Aguascalientes, México.

## Methods

### Design and Subjects

An intervention study was carried out using 3 groups of physicians, 2 of which were subjected to educational intervention strategies (CR or PBL), and a third that acted as the control group. CC was evaluated and compared both within and between groups.

### Settings

The Health Institute of the State of Aguascalientes (HISA) provides healthcare to a population of nearly 389,000 residents of the city of Aguascalientes, Mexico, which is not covered by the Social Security services. It consists of 12 healthcare clinics employing 68 general physicians. A mean of 5.6 physicians per clinic perform an average of 11.2 medical consultations per day.

### Study population, group formation, sample size and power analysis

As the HISA has no substitute physicians to cover permanent staff and since the health clinics cannot close, a random number list was generated using the EpiInfo program and used to select 38 of the 68 physicians working in the HISA. The physicians not selected covered the hours of the study participants in their clinics. Of the physicians selected for the study, 8 were not included because they were assigned to carry out different activities, or they were on vacation/leave of absence. The remaining 30 physicians were randomly assigned to 3 study groups using an electronic randomization list. A post-hoc power analysis was performed using the G Power 3.1.3 statistical program, as described previously [18]. A two-tailed Student´s t-test was used to determine whether 10 physicians per study group was a sufficiently large sample to detect statistical differences within (difference of two dependent means) and between (difference of two independent means) groups, with an alpha level set at 0.017 and the effect size (dz) calculating group parameters (mean and standard deviation of each group).

### CME interventions

The learning objectives were the same for both intervention groups: to improve the effectiveness of primary health care physicians in all three dimensions of CC in 9 priority health care programs (Hypertension, Diabetes Mellitus, Diarrheas and Acute Respiratory Infections, Prenatal Health, Nutrition, Family planning, Women’s Health, Tuberculosis and Vaccines). Both interventions ran for 4 days, lasting 8 hours per day.

In both interventions a session for each of the priority healthcare programs was planned, with the content focusing on preventive, diagnostic and therapeutic processes, as well as patient-doctor interactions. All the content was considered that were compliant with the quality of clinical healthcare guidelines established by the continuing healthcare quality program of the Mexican Health Ministry.

Both interventions were carried out in the education department at the central office of the HISA; this department had classrooms and a library with access to electronic libraries, which were used by the participants after the sessions to gather new information.

The first evaluation was carried out 3 weeks prior to the interventions the second took place four weeks post-intervention. The same instruments and data collection techniques were used to evaluate both groups.

### CR educational strategy

An educational professional with expertise in critical reading was appointed as the group tutor, and the CR intervention was developed in three phases:

1. *Planning.* An expert in each of the priority health programs gathered reading material on the issues most relevant to the competence of a general physician in a primary healthcare setting. Up-to-date information was included (original articles, reviews and healthcare programs), and the tutor acted as a consultant and supervisor.

2. *Implementation.* The sessions were overseen by the tutor with help from a program expert (Chair). Participating physicians received the reading material 8 days before the intervention, which they were asked to review and analyze. At the first session, each of the physicians presented their initial opinion of the subject matter and the core ideas they had extracted from the first reading, and they discussed how these conformed with or differed from their original point of view (based on their experience and prior knowledge). Guided by the tutor, the physicians read the material again in order to identify the key concepts and core ideas. The tutor urged the physicians to concentrate on comparing their prior knowledge and theoretical positions with the arguments presented in the reading material, assessing individually whether it reaffirmed or changed their point of view. The physicians were required to support their opinions at the end of the session and to explain their position to the group, sharing information and describing their individual analyses. The purpose of this intervention was to improve the physician’s ability to analyze reading material, systematically debate the associated issues and to enhance their capacity for statement development [19].

3. *Conclusion.* At the end of the intervention, a group report was requested by the tutor to gather the opinions of the participants regarding the lectures, the dynamics of the workshop and their tasks.

**Figure 1:**
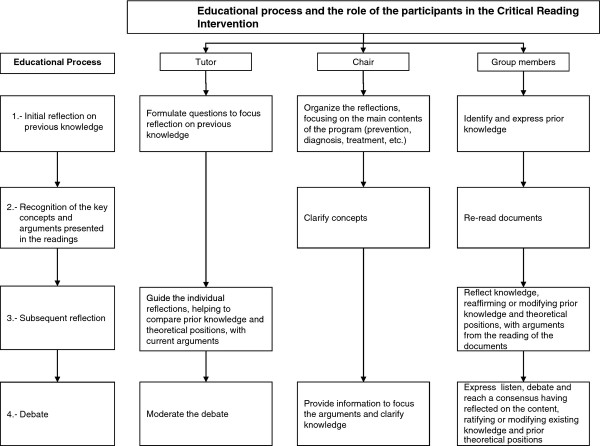
Educational process and the role of the participants in the Critical Reading Intervention.

The second and third phases were adapted from Insfran-Sanchez [20]. Figure 1 presents each of the steps in the educational process and the role played by each participant. 

### PBL educational strategies

An educational professional with expertise in PBL was appointed as the group tutor, and the intervention was designed in two phases, as described previously [21].

**Figure 2:**
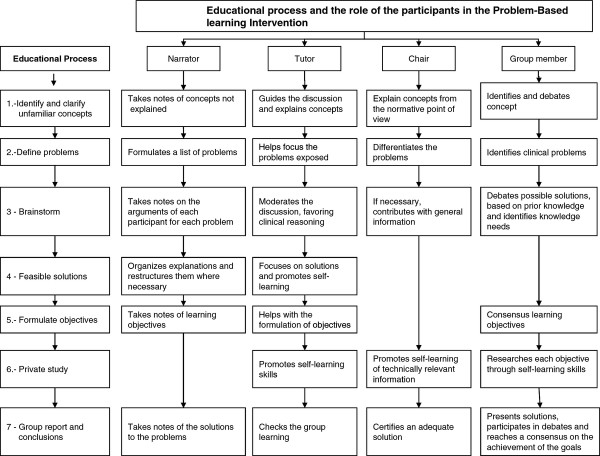
Educational process and the role of the participants in the Problem-Based intervention.

1. *Preparation.* An expert on each of the priority health programs designed a clinical case study, using information from real and simulated patients, in which physicians could identify and solve clinical problems. The case studies also incorporated the most important content that a general physician should understand to be competent in a primary healthcare setting. All physicians received the clinical case studies 8 days before the intervention, which they were asked to review and analyze.

2. *Tutorial.* The strategy ran for 4 days, 8 hours per day. The aim of this intervention was to learn to identify problems, pose questions and debate the best solutions to the clinical problems at hand, a debate that was chaired by the tutor. Each session was divided into two meetings, as described previously [22]. A session for each of the health program lasted approximately 3 hours and a half; 1 hour and a half for the discussion meeting 1 hour for individual study, and 1 hour for the concluding meeting. The strategy was carried out under the direction of the tutor with help from a program expert (Chair). At the discussion meeting, the first 5 steps proposed by Schmidt were completed [23] and the physicians analyzed the problems posed in each clinical case study. After this meeting and prior to the following meeting, the physicians had time for individual study (step 6). In the concluding meeting (step 7), a physician from the group drafted a report on the intervention, describing the participants´ experience of the presentation and dynamics of the workshop, and the resolution of the tasks involved. Figure 2 presents each of the steps in the educational process and the role played by each participant. 

### Control group

This group received no educational intervention, nor any information or documentation relating to priority health programs, although they were evaluated in the same manner as the intervention groups.

### Data collection

Information on the physician’s age, sex, seniority and time since medical graduation was collected using a structured questionnaire.

The Cognitive Dimension was evaluated by a self-administered questionnaire completed by each physician in their office. The theoretical content of the questionnaires was reviewed by six clinical experts and the structure was reviewed by three researchers. For each priority health program clinical cases were presented, generating 70 items with one best answer Multiple Choice Questionnaires (MCQs). An example of clinical cases with questions is presented in Appendix A.

Information from patient files was used to evaluate Habitual Behavior using a data compilation form with a check list. This form was designed in accordance with the quality of healthcare clinical guidelines established by the continuing healthcare quality improvement program of the Federal Ministry of Health for 4 of their priority health programs (Hypertension, Diabetes Mellitus, Diarrheas and Acute Respiratory Infections, Prenatal Health). This served as the basis to compile the forms for the Nutrition, Family planning, Women’s Health, Tuberculosis and Vaccines programs. These forms were revised by 6 clinical experts and 3 researchers, and a total of 69 items from the 9 programs were generated, generating a register of the performance of a specific action with dichotomous answers. From the daily patient register of the previous days 2 patients per physician were identified or selected for each priority health program, a total of 18 patients per physician. Thus, each physician was evaluated on 138 items.

The Affective Dimension was evaluated by interviewing patients using a validated questionnaire developed by Smith and Falvo, which measures patient perceptions of the patient-doctor interaction [24]. This approach has demonstrated adequate internal consistency (Cronbach’s Alpha = 0.80 [25,26]), and was adapted and translated into Spanish. The Spanish questionnaire also demonstrated adequate internal consistency (Cronbach’s Alpha = 0.90). Nineteen items were rated on a five-point Likert scale of agreement (1 to 5) that ranged from “strongly agree” to “strongly disagree”. The questionnaire was completed in the waiting room of the healthcare clinics directly after patient consultation. Five surveys were administered per physician.

Each instrument was applied by the principal investigator 3 weeks before and 4 weeks after the intervention. The quality of the data was evaluated immediately after collection and the EpiInfo Ver. 6.04 program was used to create a database.

### Statistical analysis

For each physician the Cognitive dimension was scored as the number of correct answers for the 70 items. From the 18 patient clinical files per physician, the Habitual Behavior dimension was scored as the mean of items for which there was a register in the patient’s clinical files of the performance for each of the 138 actions. The scores for the Affective dimension were calculated for each patient as the mean of the 5 point Likert value for the 19 items. From the 5 patients, a mean score was then calculated per physician.

For quantitative variables, measures of the central tendency and the dispersion were used to summarize the data. Variables with categorical scales were analyzed using percentages and absolute frequencies. To identify the differences between the three groups at the beginning of the study, qualitative variables (sex) were assessed using a chi-squared test, and quantitative variables (age, seniority and time since medical graduation) with a 1-way ANOVA. Similarly, a 1-way ANOVA was also used to compare pre-intervention scores within each of the 3 dimensions per group. In both cases, a *p* value of > 0.05 was considered statistically significant. A paired *t* test was used to identify differences between the means before and after the intervention for each group, and for each dimension. As each dimension represents a dependent variable, the alpha level was set at 0.017 (0.05/3 = 0.017).

A 3 (groups) x 2 (times) ANOVA was performed for each dimension, with groups as between and times as within variables, and of their interaction, to compare the pre- and post-intervention scores for each group. Data processing and analysis was performed using the Statgraphics Centurion XV statistical package.

## Results

Of the 30 GPs included in this study, 2 were removed from the analysis as they did not adequately complete the course (*i.e.:* they did not attend at least 90% of the sessions). Thus, 28 GPs completed the course, 9 in each intervention group and 10 in the control group, of which 46.4% were women. The mean age of the GPs was 44.01 (+/−5.94) years and their seniority in the HISA ranged from 1 to 28 years. Time since graduation from medical school ranged from 14 to 29 years. The majority of GPs were over 40 years of age and the average time spent working at the HISA following graduation was 4 years. No significant differences in any of these variables were detected between groups (Table 1).

**Table 1:** Group characteristics

	**Critical Reading**	**Problem-Based Learning**	**Controls**	**Total**
Age*	44.55	45.44	44.70	44.01
	(± 5.98)	(± 4.39)	(± 6.88)	(± 5.94)
Seniority*	15.55	14.11	10.80	13.06
	(± 5.65)	(± 3.06)	(± 7.37)	(± 5.72)
Time Since Graduation (yrs)*	17.88	17.88	18.40	17.9
	(± 5.62)	(± 2.71)	(± 5.29)	(± 5.21)

* F test (p >0.05).

### Initial evaluation

Low scores were obtained in the cognitive dimension for all three groups, with the mean ranging from 43.11 in the CR group to 45.66 in the PBL group (maximum score = 70). In the habitual behavior dimension, the mean scores ranged from 52.3 in the control group to 57.44 in the CR group (maximum score = 138), whereas in the affective dimension, the CR group had the highest median scores (4.39). The negative score for skewness in the affective dimension of the PBL group indicates a shift of the peak to the right of the normal distribution, the negative kurtosis indicates that there are more scores in the tails that in the peak, 55.6% of the 9 physicians obtained the best possible score (5 points in the Likert scale). There were no significant differences between these groups in any of the three CC dimensions (p >0.05; Table 2).

**Table 2:** Pre-intervention scores for each of the three dimensions of clinical competence in the three study groups

	**Mean**	**Standard deviation**	**Skewness**	**Kurtosis**	**F-test**	**p-value**
Cognitive Dimension						
Critical Reading	43.11	8.49	0.58	1.08		
Problem-Based Learning	45.66	6.94	- 0.47	0.17	0.25	0.78
Controls	43.90	8.13	1.27	0.48		
Habitual Behavioral Dimension						
Critical Reading	57.44	13.62	- 0.17	- 0.16		
Problem-Based Learning	53.77	13.80	0.25	0.42	0.30	0.74
Controls	52.30	16.66	- 0.95	0.84		
	**Median**	**Lower-upper quartile**	**Skewness**	**Kurtosis**	**Kruskal-Wallis**	**P-value**
Affective Dimension						
Critical Reading	4.39	4.23-4.58	- 0.97	- 0.34		
Problem-Based Learning	4.26	4.08-4.68	−2.87	- 3.69	0.53	0.76
Controls	4.26	3.8-4.54	- 0.92	−0.13		

### Pre versus post-intervention scores for each group

Within the cognitive dimension, significant differences between pre and post-intervention scores were only detected in the PBL group (p > 0.017), in which a 9.22 point increase was observed. A post-hoc power analysis for this PBL group revealed that a sample of 9 physicians provided a power of 98% in detecting statistical differences for the effect size given by the mean scores within this group (Table 3).

**Table 3:** Pre- and post-intervention scores for each study group in the three dimensions of clinical competence

		**n**	**Mean**	**95% C.I.**		**S.D.**	**t-test**	**p-value**	**Power**	**Effect size dz**
				**Inf**	**Sup**					
Cognitive Dimension										
Critical Reading	Pre	9	43.11	39.28	46.94	8.49	−2.97	0.017	0.51	0.98
	Post	9	51.55	47.81	55.29	8.11				
Problem-Based Learning	Pre	9	45.67	41.83	49.49	6.95	- 5.77	0.000	0.98	1.93
	Post	9	54.89	51.15	58.63	5.49				
Controls	Pre	10	43.90	40.26	47.54	8.13	−2.24	0.051	0.31	0.71
	Post	10	49.90	46.35	53.45	8.92				
Habitual Behavioral Dimension										
Critical Reading	Pre	9	57.44	50.24	64.65	13.62	−7.85	0.000	0.99	2.61
	Post	9	85.00	79.71	90.28	5.31				
Problem-Based Learning	Pre	9	53.78	46.57	60.98	13.81	−6.60	0.000	0.99	2.21
	Post	9	82.33	77.05	87.62	6.20				
Controls	Pre	10	52.30	45.46	59.14	16.67	2.51	0.037	078	1.19
	Post	10	74.20	69.18	79.21	16.43				
Affective Dimension										
Critical Reading	Pre	9	4.37	4.11	4.64	0.26	5.20	0.000	0.96	0.48
	Post	9	4.77	4.66	4.87	0.14				
Problem-Based Learning	Pre	9	4.16	3.90	4.42	0.58	2.66	0.007	0.82	1.18
	Post	9	4.76	4.65	4.87	0.25				
Controls	Pre	10	4.17	3.92	4.42	0.47	3.14	0.011	0.81	0.14
	Post	10	4.79	4.63	4.83	0.25				

The control group was the only group in which no significant differences were observed in the habitual behavioral dimension (p > 0.017). Indeed, post-hoc power analysis revealed that the sample size for the Critical Reading and the PBL group (9 physicians per group) had a statistical power of 99% to detect statistical differences for the effect size given by the mean scores within each group (Table 3).

Significant differences in the pre- versus post-intervention scores for the affective dimension were detected for all three groups. The post-hoc power analysis revealed the sample size of each group to be adequate, with a statistical power of over 80% (Table 3).

### Evaluation of pre versus post-intervention scores between 3 groups

In all three dimensions there were significant differences within the groups in the pre- versus post-intervention scores, yet no significant differences were observed between groups. No interaction between pre- and post-intervention differences was detected in any of the groups for any dimension (Table 4). A post-hoc power analysis revealed that the sample size was insufficient to detect statistical differences in each of the groups for the effect size given by the mean scores between each group after the interventions.

**Table 4:** 3 x 2 ANOVA of groups, times and their interaction

	**F-Ratio**	**Df**	**P-Value**
Cognitive Dimension			
Between Groups	1.03	2	0.3662
Within Times	14.29	1	0.0004
Interaction	0.22	2	0.8007
Groups * Times			
Habitual Behavioral Dimension			
Between Groups	1.81	2	0.1735
Within Times	55.72	1	0.0000
Interaction	0.37	2	0.6954
Groups * Times			
Affective Dimension			
Between Groups	0.49	2	0.6172
Within Times	21.83	1	0.0000
Interaction	0.31	2	0.7349
Groups * Times			

## Discussion

In this study, we have evaluated the CR and PBL based improvement in CC among groups of general physicians in Mexico. The low CC observed in the initial evaluation for the cognitive and habitual behavior dimensions may be due to physicians not staying up to date on the latest advances in their field, given their heavy workload [27] and working conditions [5]. Indeed, the isolated nature of a general physician´s work provides little opportunity for regular discussion of clinical matters [28]. Other personal factors, such as a physician’s motivation and attitudes, may also be reflected in their CC [29].

In relation to the priority health programs, a low CC in the cognitive dimension has been reported previously among family physicians in Mexico City using similar methods [5,27]. Moreover, similar results were also obtained in New Zealand [28], and low CC in the habitual behavior dimension was identified in standardized patients and audits of the clinical histories of primary care physicians in Spain [6].

The high CC scores observed in the affective dimension may be due to GPs assuming a paternalistic doctor-patient relationship, acting as the “good guy” in the predominant relation model proposed by Epstein [30]. In contrast to our results, deficiencies in the doctor-patient relationship have been reported in other studies [31,32], conflicting findings that may be explained by differences in population expectations not identified by the methods used here.

We detected no significant improvement in the cognitive dimension after the CR intervention. By contrast, previous studies of medical students [13] and junior resident doctors [33] reported cognitive improvements following a CR intervention, a discrepancy that may reflect the type of knowledge evaluation used in each study. Here, we used questions relating to clinical case studies that tested the participant´s application of knowledge, as opposed to the comprehension or interpretation of concepts and principles tested elsewhere. Moreover, the sample size in our group did not had the sufficient power to detect a significant median size difference in pre- versus post-intervention scores, which may require a larger sample in the CR group.

The CR group exhibited a significant increase in the habitual behavior score. Although the CR intervention focuses on the acquisition of knowledge, the improvements in habitual behavior were not unexpected. In one of the stages of the strategy, the tutor and participants discuss the incorporation of knowledge in their working environment, mainly focusing on documents relating to healthcare programs. This strategy provides a better understanding of the actions to be recorded in patients’ clinical files (habitual behavior dimension).

As CR does not modify the affective dimension, this parameter was not evaluated in other studies. Nonetheless, we observed a significant increase in the affective dimension that may be explained by contamination from the PBL group (which does have an emotive focus), since both groups share a small clinical working area.

After the intervention in the PBL group, we observed an increase in CC in all three dimensions. PBL is a strategy that promotes learning through guided discovery, increasing medical knowledge and reinforcing procedural skills, thereby increasing the probability that the GP will adequately complete the clinical files of their patients. PBL ultimately aims to provide a “Habitual Behavior” to improve therapeutic diagnostic procedures and skills [17]. A meta-analysis has demonstrated the positive effect of PBL strategies on knowledge application [34]. Indeed, our results for the PBL group matched those previously reported for a group of physicians that improved their knowledge and their therapeutic diagnostic skills for the treatment of headache patients [11]. Increased motivation has also been reported in groups subjected to PBL strategies [35], as well as improved communication and interpersonal skills [36], which may help identify affective problems in the physician-patient relationship. Taken together, these effects contribute to an improvement in the affective dimension in the post-intervention evaluation.

After the intervention, the control group exhibited differences in the affective dimension, yet no improvement in knowledge or habitual behavior since they had not been exposed to any educational intervention. The increase in the affective dimension may have been due to a Hawthorne effect [37] whereby physicians who are aware that they are being observed pay special attention to their clinical activities, mainly by improving their relationships with patients.

Since there were no significant differences between groups, it is not clear which of the two educational strategies is more efficient in improving the CC of primary healthcare physicians. Indeed, none of the groups were sufficiently large as to detect differences in effect (*i.e.:* in the differences in the mean scores between groups after the intervention). Based on the mean and S.D. post-intervention values for the CR and PBL groups in the thee CC dimensions, the sample size required to detect a significant difference would be 77 physicians per group, or a total of 154 physicians. In the city of Aguascalientes, the HISA employs only 68 physicians and thus, it will not be feasible to conduct such a study in this context.

The use of small groups is common in comparative studies of educational methods [10]. Indeed, small groups may be preferable to plenary sessions to perform CR [20], and small group discussions in PBL have a positive effect on the intrinsic interest of those involved, producing better cognitive and motivational content [8]. Nevertheless, restrictions on sample size in these studies are common. In our study, we used small groups but with a broad evaluation in terms of quantity and content, which allows more consistent CC evaluation in a priority program. However, as in most studies with small groups it would be better to increase the number of groups and participants.

A randomized controlled trial has been conducted to investigate the utility and efficacy of guideline dissemination in asthma management, using PBL with a small-group (23 family physicians) and a didactic lecture session (29 physicians), testing knowledge (nine items), skills (seven items) and attitudes (nine items) [38]. In agreement with the present findings study, both groups exhibited significant improvements in the knowledge, skills and attitudes dimensions. Performance varied over time, as evident through the significant main effect for time, although no differences were detected between PBL and more didactic learning sessions in terms of facilitating knowledge gain, knowledge retention, or changes in attitude regarding asthma management. A randomized controlled trial of 118 trainee occupational health physicians compared the effectiveness of PBL to lecture-based learning [39], as in our study, performance scores increased significantly in both groups, although no significant differences were observed between groups. It should be noted that these studies [38,39] used lecture-based formats in the control groups, whereas in this study, both PBL and CR were based on a collaborative active educational approach, which should also be considered when discussing the failure to detect significant differences.

Different methods are used to evaluate improvements in CC as a result of continuing medical education. Thus, evaluating the Integral CC alone may fail to identify improvements in specific dimensions, as described in previous studies of medical students and resident doctors [13,33,40].

### Limitations

The present study did not have sufficient power in terms of sample size to determine which of the two interventions was more effective in improving CC. However, the largest and most significant improvement in all three dimensions of CC was detected in the PBL group.

In the present study, we used different methods for each of the CC dimensions, each with their own limitations. For example, the Habitual Behavior dimension was evaluated using information from the patient’s clinical files, which only analyzes clinical skills within a limited range (omitting examination or patient management skills). Moreover, this information generally only demonstrates that the physician has registered a specific action, without indicating whether it was performed or understood correctly. This particular approach was used as this is the strategy recommended by the ministry of health in México to evaluate a physician’s performance. The measure used to evaluate the affective dimension determines the patient’s relative satisfaction. As in most measures of satisfaction, a ceiling effect is observed in the PBL group, reflecting an overestimation of this dimension. The values for skewness (−2.87) and kurtosis (−3.69) in this dimension for the PBL group indicates that most of the scores are shifted to the upper value of the Likert scale, 55.5% of the 9 physicians obtained the best possible score (5 points in the Likert scale) showing an important ceiling effect. Neither the CR nor the Control group showed a floor or a ceiling effect. Increased motivation and improvement of communication and interpersonal skills had been reported in groups subjected to PBL [35,36] therefore we cannot rule out that the effect observed is a true effect of the intervention. Nevertheless in future studies of the affective dimension of CC using this questionnaire, a seven point Likert scale, as well as an increment in the sample size may give results with higher variability.

Some contamination occurred between the intervention groups and the control group, due to their sharing of common work spaces in small clinics. In future studies, this effect may be prevented by randomizing the clinics participating, whereby all physicians receive the same educational intervention rather than randomizing physicians into specific the study groups.

Potentially confounding characteristics of the educational process were not analyzed, including the time physicians dedicated to their tasks outside of the sessions, the quality of the educational resources used in the strategies, the intrinsic motivation of the physicians, and their satisfaction with the learning experience. However, the consistency of our results with those of other studies in which these variables were analyzed [38,39] suggests that any effect of these variables was equivalent in all groups.

## Conclusions

While we found no significant differences between study groups, we can conclude that the PBL strategy led to the greatest increases in post-intervention scores and that it was the only strategy that produced a significant difference pre- versus post-intervention in our group of physicians for all three CC dimensions. These findings are in agreement with a previous study that reported no improvement in CC scores using PBL when compared with other conventional methods such as CR [41]. However, a study of graduate physicians reported that PBL was more effective than a lecture format in improving scores in the cognitive dimension [11]. PBL is an educational strategy that can be applied according to the competence of the study participants, taking into account differences in educational training, continuous education and health institutions.

## Appendix A

Sample of cases and questions for one of the priority health programs (Diabetes Mellitus).

### PREVENTION

Case 1: A 26-year-old man, with family history of a father with DM2 under medical treatment. The patient works as a clerk in a government office. He does not practice any physical activity after work. He has been a smoker since he was 15 years old smoking up to 7 cigarettes daily. He takes between 5 to 7 alcoholic drinks twice a week. He assists to a regular medical checkup in his health center. PE: BMI 32.7, BP 118/82, HR 81 bpm. Acanthosis nigricans in neck grade 1.

1. The General Practitioner informs about the presence of the following risk factor for DM2.

a) Age, sedentary and smoking habit.

b) Age, sex and family history of diabetes.

c) Sex, Acanthosis nigricans, smoking habit.

d) Family history of diabetes, obesity and sedentary.

e) Family history of diabetes, smoking habit and age.

2. As a General Practitioner, you would recommend the following screening strategy.

a) This patient does not require screening test because he is less than 45 years old.

b) Inform the patient to return in 3 years for a capillary blood glucose test.

c) Request a HbA1c test.

d) Request a fasting capillary blood glucose test.

e) Request an oral glucose tolerance test.

3. The result of the screening test was: 90 mg/dL: What is the course of action to follow?

a) Repeat the capillary blood glucose test.

b) Repeat the capillary blood glucose test in 3 years.

c) Request a fasting plasma glucose test.

d) Request an oral glucose tolerance test.

e) c) Request aHbA1c test.

### DIAGNOSIS

Case 2: A 35-year-old woman, with family history of paternal grandfather with DM2. The patient smokes 6 cigarettes daily, takes alcoholic drinks every 15 days (approximately 4–8 cups) and has a sedentary life style. Her General Practitioner asks for laboratory tests having the following results: glucose 116 mg/dL, Total Cholesterol 286 mg/dL, LDL 160 mg/dL, HDL 34 mg/dL, Triglycerides 295 mg/dL. PE: BMI 33.5, BP 130/90, HR 78 bpm.

1. What would it be the diagnosis based on previous information?

a) Obesity, high blood pressure, and type 2 Diabetes Mellitus.

b) Obesity, glucose intolerance, and mixed dyslipidemia

c) Obesity, high blood pressure, and glucose intolerance

d) Obesity, mixed dyslipidemia, and altered fasting glucose level

e) Overweight and type 2 Diabetes Mellitus.

2.-What others laboratory test would you request to complete your diagnosis?

a) Repeat a fasting plasma glucose test.

b) Request an oral glucose tolerance test.

c) Request a HbA1c test.

d) Request a General Urine test.

e) Request a fasting plasma glucose test.

3. The patient returns with the results of laboratory tests having fasting plasma glucose of 109 mg/dl, and 2-hour glucose of 190 mg/dL. What is the diagnosis?

a) Type 2 Diabetes Mellitus.

b) Type 1 Diabetes Mellitus.

c) Results are normal.

d) Altered fasting glucose.

e) Impaired glucose intolerance.

### TREATMENT

Case 3: Male 46 years old. Patient’s father was diabetic since 55 years old with history of ischemic heart disease. Patient’s mother has essential hypertension. The patient has been smoking since 20 years old, a pack daily for the last 2 years. His life style is sedentary. He attends public health services with a report of fasting plasma glucose test of 110 mg/dL. PE; BMI 37.4. BP 142/80. You request an oral glucose tolerance test reporting a fasting plasma glucose of 104 mg/dl, and 2-hour glucose of 138 mg/dL. 

1. Is the patient candidate to start medical treatment as prevention for DM2?.

a) Yes, he has pre-diabetes.

b) Yes, he is less than 60 years old and has pre-diabetes.

c) No, he is older than 40 years.

d) No, the initial preventive management is changing his lifestyle.

e) No, he has normal fasting glucose.

2. Does this patient need any other management?

a) Dilated eye exam to screen for diabetic retinopathy.

b) Cardiac stress test.

c) Stop smoking.

d) Chest radiography

e) Vitamins B supplements.

## Abbreviations

CR: Critical Reading; PBL: Problem Based Learning; BPHS: Basic Package of Health Services; GP: General Practitioner; CC: Clinical Competence; CME: Continuing Medical Education; CAE: Collaborative Active Education; MCQs: Multiple Choice Questionnaire; HISA: Health Institute of the State of Aguascalientes.

## Competing interests

The authors have no competing interests to declare.

## Authors’ contributions

JGO and CAPA participated in the study design, data collection, statistical analysis, data interpretation, and the drafting of the manuscript. YSB collaborated in data collection, statistical analysis and the drafting of the manuscript. JVM and MGA participated in the implementation of the educational strategies, data collection and the drafting of the manuscript. All authors read and approved the final manuscript.

## Pre-publication history

The pre-publication history for this paper can be accessed here:

http://www.biomedcentral.com/1472-6920/12/53/prepub
